# Monatsschrift Für Ohrenheilkunde

**Published:** 1872-01

**Authors:** 


### MONATSSCHRIFT FUR OHRENHEILKUNDE.

We have received, through our friend, Dr. A. W. Calhoun, of Newnan, Georgia, now in Berlin, Prussia, a late number of the Monatsschrift für Ohrenheilkunde, and are indebted to Dr. Ch. Raushenberg, of this city, for the following notice of its contents:

The November number of a Monthly for Aural Medicine, published at Berlin, and edited by Professors Voltolini, Gruber, Rûdinger and Dr. Weber, respectively of Breslau, Vienna, Munich and Berlin, presents at first sight rather a meagre exterior for a German monthly; but its thoroughly scientific contents, on the diseases of the organs to which it is devoted, compensate the reader richly for the modesty of its appearance and dimensions.

An article under the heading of "Otitis Intermittens," by Dr. F. E. Weber, contains the history of several cases, of which we give a condensation of one, based upon the evidently very exact self-observations of the sufferer, Dr. Harden, of Berlin, who was treated by Dr. Weber.

The disease commenced on September 10, late in the afternoon, after a walk of one and a half hours duration in very windy weather, with a singing and, immediately afterward, buzzing noise, the sensation of obstruction and a diminution of hearing in the right ear. A slight nasal and pharyngeal catarrh, of a week's standing, induced the supposition of a transition of this condition through the tuba eustachia. Chilliness, headache, vertigo, extreme sensation of fatigue, gnawing pains and pulsating noises, and, from eleven o'clock, P. M., furious boring, beating, and sometimes pressing, pains, radiating toward the right eye, the inferior maxilla and the shoulder, with painful sensations when closing the mouth firmly or opening it wide, continued until nine A. M. next day. The general malaise, headache, vertigo, loss of appetite, pain in the right maxillary articulation and the right orbitus, stiffness of the neck, slight rigors for several evenings in succession, with marked inclination to perspire when in bed, continued until the 18th, when patient could leave his room and walk about; but considerable diminution of hearing remained. The speculum showed hyperæmia of the external meatus and light vascular injection of the tympanum. The introduction of the tympanum-catheter, and blowing of air into the cavity of the middle ear, brought course râles to the perception of the patient, and the removal of a small quantity of tenaceous turbid fluid, by aspiration through the catheter, caused an increased resonance of the patient's voice, a sensation of relief, and marked increase of hearing. This process was repeated on the 25th ; but, as the improvement obtained was only transient, the tympanum was perforated and a small quantity of muco-purulent fluid removed. Repeated catheterization, the air-douche, and hydriodate of potass externally and internally, were used, and once more a small quantity of the same fluid removed, but without any material improvement of hearing, even after the catarrhal symptoms had disappeared. On October 9th, after a walk of one and a half hours duration, moderate pains in the ear, with pulsating noises, chilliness, loss of appetite and insipid taste set in, and after going to bed a sensation of oppression about the chest, with chilly sensations, gave way to a light fever and moderate, but marked general perspiration afterwards. Next day, with exception of abnormal sensations in, and general hyperæsthesia of the ear, the patient was well enough. Towards night a repetition of yesterday's symptoms, with intense pains like those at the commencement, radiating to the right eye, maxilla and shoulder, with a sensation of contraction of the masseter, and an absolutely stiff neck, set in, followed again, late in the night, by chilliness, fever and perspiration in regular succession. The 11th and 12th presented the same occurrences as the two previous days. Quinine, in full doses, taken late on the 12th, prevented a return of the same symptoms next day. It was repeated on the two following days, and it caused a cessation of all abnormal sensations about the affected ear, and on the 18th the cavity of the tympanum was free of secretion and the hearing almost entirely restored.

This case is considered quite suggestive of new considerations for the genesis of some aural derangements, and in full proof of views formerly expressed by Dr. Weber, to the effect that many lesions considered to be middle ear catarrh are referable to primary affections of the presiding nerves. Weber is of the opinion that in the above related case no common inflammatory process was going on, no otitis media acuta was on hand, and that the rigors were not the result of pyæmic infection from a suppurating hearth (point of infection), because: 1st, the inspection of the tympanum and the condition of the fluid removed by aspiration through the catheter, disproved the existence of fresh or decomposed pus within the tympanum; 2nd, the apyrexia was as specifically developed as the specific phenomena of the paroxysms, and the effect of the quinine furnished decisive evidence of the intermittent character of the disease.

He considers these cases similar in their nature to those of opthalmia intermittens, and interprets the morbid process they represent thus: after the irritative effect of the malarious poison has exhausted itself at the initiation of the disease by a severe paroxysm of pain, involving simultaneously all the branches of the trigeminus with radiation to the connecting nerves, it continues its manifestations for some time only in the form of a vaso-motor neurosis, the effect of which causes the injection of the tympanum and the recurring secretion of muco-purulent discharge within the cavity.

These exudations could also be explained by the possible paralytic dilatation of the tympanic and intra-auricular capillaries, the result of reflux action from excessive irritation of the sensitive nerve fibres.

The second intense fever explosion, which localized itself again prominently in those parts of the ear supplied by the third branch of the trigeminus, introduced the plain intermittent stage of the disease, in which not only the vaso-motor, but also the sensitive and motory fibres of that branch of the trigeminus were affected.

In a similar manner, other intermitteut and non-intermittent neuroses of the sensitive and motory nerves of the acoustic apparatus present the appearance of catarrhal affections of the middle ear, of changed secretions of the external meatus, while, in reality, they are only symptoms of acute and chronic secretory neuroses, in the same way in which certain pernicious purulent middle-ear inflammations bear plainly the character of neuro-paralytic otitis.

Professor R. Voltolini, in an article "On the Ossicula of the Ear of Egyptian Mummies," concludes that they are, in every particular of size and shape, like the ossicula of the present day; hence, furnish no evidence of difference of race, or any change of organization, which, according to Darwin's doctrines, might be expected to have manifested itself to some extent within a period of four thousand years, the lowest estimate for the age of those mummies from which he obtained his specimens.

Two able articles, one by Dr. Gruber on the anomalies of the tension of the tympanum, and one by Dr. Weber on the pathology and treatment of the diseases of the muscles of the middle ear, with a literary report of late publications on aural diseases, embrace the contents of this number.

